# A Comparative Analysis of Feeding Practices and Oral Immunity in Infants

**DOI:** 10.3390/medicina61061114

**Published:** 2025-06-19

**Authors:** Amal Mohamad Husein Mackawy, Fay Saleh Alturky, Amal Hussain Mohammed, Basmah F. Alharbi, Mohsina Huq, Afshan Zeeshan Wasti, Mawahib Alhag Ali Ahmed, Hajed Obaid Abdullah Alharbi

**Affiliations:** 1 Department of Medical Laboratories, College of Applied Medical Sciences, Qassim University, Buraydah 51452, Saudi Arabia; 411201893@qu.edu.sa (F.S.A.); ama.ali@qu.edu.sa (A.H.M.); m.huq@qu.edu.sa (M.H.); a.wasti@qu.edu.sa (A.Z.W.); maw.ahmed@qu.edu.sa (M.A.A.A.); 2 Department of Basic Health Sciences, College of Applied Medical Sciences, Qassim University, Buraydah 51452, Saudi Arabia; b.alwahbi@qu.edu.sa

**Keywords:** infant health, infant feeding, Interleukin-17, cytokines, oral microbiome, breastfeeding, formula feeding, epithelial cells, oral bacterial load, oral bacterial infection, pediatric buccal health

## Abstract

*Background and Objectives*: Infant feeding practices play a crucial role in shaping the oral microbiome, modulating inflammatory responses, and maintaining epithelial health during the first year of life. Breastfeeding promotes the growth of beneficial bacteria and supports a diverse, stable microbial community. In contrast, formula feeding is associated with increased colonization by potentially pathogenic bacteria, such as Staphylococcus and Escherichia coli, which may elevate the risk of infections, oral diseases, and inflammation. This study investigates the effects of breastfeeding versus formula feeding on oral bacterial growth, epithelial cell integrity, and interleukin-17 (IL-17) expression in infants aged 1–12 months. *Materials and Methods*: A total of 60 infants (30 breastfed and 30 formula-fed) were recruited from pediatric clinics in the Qassim region. Microbial cultures quantified bacterial colony-forming units (CFUs), and epithelial cell morphology was assessed through the microscopic analysis of mucosal scrapings. IL-17 concentrations were quantified from the oral mucosa through enzyme-linked immunosorbent assay. Statistical analyses, including t-tests and chi-square tests, compared bacterial loads, IL-17 levels, and indicators of epithelial health between groups. Adjustment for potential confounders was achieved through multivariate statistical analysis. *Results*: Formula-fed infants showed significantly higher IL-17 levels than breastfed infants (*p* < 0.001), indicating a stronger pro-inflammatory profile. Breastfed infants exhibited lower inflammation, improved epithelial health, and reduced cellular debris compared to formula-fed infants, who had higher bacterial loads. A significant correlation was found between epithelial health and bacterial clustering, with clearer epithelial cells associated with lower bacterial colonization. *Conclusions*: Formula feeding was associated with increased salivary IL-17 levels, greater bacterial colonization, and compromised epithelial integrity, indicating a heightened pro-inflammatory state and potential vulnerability to mucosal irritation or infection. Breastfeeding appeared to confer protective effects by promoting healthier microbial balance, epithelial integrity, and reducing inflammatory responses. These findings underscore the immunological and microbial benefits of breastfeeding in supporting oral health during infancy.

## 1. Introduction

Early-life nutrition plays a critical role in shaping the infant microbiome and immune system, with breastfeeding recognized as a key determinant of neonatal health [1,2]. Human milk provides not only essential nutrients but also bioactive compounds, such as immunoglobulins, cytokines, and human milk oligosaccharides (HMOs), which promote colonization by beneficial microbes and support immune maturation [3,4].

Epidemiological studies link exclusive breastfeeding to reduced risks of gastrointestinal and respiratory infections, lower infant mortality, and long-term protection against obesity and chronic diseases, with correlation with higher IQ scores in childhood, and a 36% lower risk of sudden infant death syndrome (SIDS) [5,6,7,8,9,10,11]. Conversely, formula feeding has been associated with an increased risk of oral health problems and disruptions in microbiota development [11], which may increase the risk of infections and inflammation [2,7,12].

The interactions between the microbiota and neonatal oral epithelium help regulate mucosal balance and may have long-term implications for health [9]. While much attention has focused on the gut microbiome, the oral cavity serves as a critical early site for microbial colonization and immune education [9,10].

Epithelial cells in the oral mucosa form a frontline barrier and actively participate in immune signaling. Oral exfoliative cytology serves as a valuable diagnostic tool, with cytomorphometric analysis offering a noninvasive method for detecting inflammation and sensitization [13,14].

The cytokine interleukin-17 (IL-17), primarily secreted by Th17 and γδ T17 cells, is essential for mucosal defense and epithelial barrier integrity in early life [15]. Disruptions in early microbial colonization (e.g., from formula feeding or cesarean delivery) may lead to altered IL-17 responses, increasing susceptibility to oral infections, mucosal inflammation, or even systemic immune dysregulation [16,17].

However, the impact of infant feeding practices on the oral microbiota–IL-17–epithelium axis remains poorly understood. Specifically, how breastfeeding versus formula feeding influences oral microbial communities and downstream mucosal immune responses has yet to be clearly elucidated.

This study aims to compare salivary IL-17 levels, bacterial colonization, and epithelial integrity of the oral cavities between breastfed and formula-fed infants during the first 12 months of life, to assess the impact of early feeding practices on oral mucosal immunity, microbial colonization patterns, and inflammation. Additionally, it aims to analyze the morphological changes and turnover rates of epithelial cells in the oral cavities, assessing their potential impact on oral barrier integrity and disease susceptibility. Expanding knowledge on how early feeding choices shape the oral microbiome can provide valuable insights for clinical guidelines and infant nutrition recommendations.

## 2. Methodology

### 2.1. Study Design

This cross-sectional study evaluated differences in oral bacterial growth and epithelial cell changes between breastfed and formula-fed infants. The study was conducted over four months, from August 2024 to December 2024, in pediatric clinics and community centers within the Qassim region, KSA. The recruitment period was selected based on logistical feasibility and clinic availability during the research funding cycle. While this four-month window falls within late summer to early winter in Saudi Arabia, we acknowledge the possibility of seasonal variation in infection rates. The study design allowed for a comparative analysis of microbial composition and epithelial integrity in the oral cavities of breastfed and formula-fed infants.

### 2.2. Study Sample

The study population consisted of infants aged 1 to 12 months who were visiting participating pediatric clinics. The study employed a clinic-based recruitment approach, targeting infants presenting at pediatric outpatient clinics across the Qassim region. The sample size was determined through power analysis using the G*Power program, software version 3.1 (Heinrich Heine University, Düsseldorf, Germany), ensuring statistical significance with a significance level of 0.05, a power of 80%, and an effect size of 0.3. Based on these parameters, the required sample size per group ≈ is 26 infants, and the minimum total sample size ≈ is 52 infants. A final recommended total sample size of 60 infants (30 per group) was used: 30 breastfed infants (Group 1) and 30 formula-fed infants (Group 2) [18].

#### Participant Flow and STROBE Compliance

**Figure 1:**
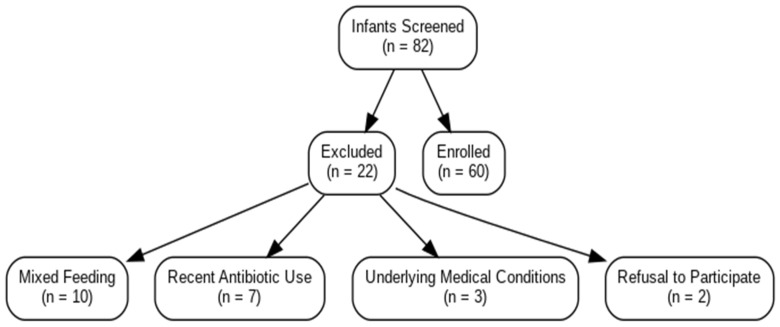
Participant flow diagram in accordance with STROBE guidelines.

A participant flow diagram has been added in accordance with STROBE guidelines to visually represent the number of infants screened (n = 82), excluded (n = 22), and finally enrolled (n = 60). Reasons for exclusion included mixed feeding (n = 10), recent antibiotic use (n = 7), underlying medical conditions (n = 3), and refusal to participate (n = 2) (Figure 1).

#### 2.2.1. Inclusion Criteria

- Infants aged between 1 and 12 months.
- Exclusively breastfed or formula-fed infants (for at least three months before enrollment).
- Healthy infants with no known underlying medical conditions or congenital anomalies.
- Parents/guardians who provided informed consent for participation.

#### 2.2.2. Exclusion Criteria

- Infants with known oral health issues, including oral infections, congenital defects affecting oral development, or diagnosed immunodeficiencies.
- Infants receiving both breast milk and formula (mixed feeding).
- Infants on antibiotic treatment within one month before sample collection.
- Infants with special nutritional needs or metabolic disorders.

### 2.3. Participant Consent and Data Collection

The enrollment of infants proceeded only after informed written consent was provided by their parents or legal guardians. A structured questionnaire was administered to parents or guardians to gather demographic information, infant feeding history, and general health status. All data were collected confidentially and used solely for research purposes, following ethical guidelines. A combination of structured parental questionnaires and biological sample collection was utilized to gather comprehensive data on feeding practices and oral health. The laboratory personnel involved in microbial culture analysis, ELISA assays for IL-17 quantification, and cytological evaluations were blinded to the infants’ feeding group assignments.

The procedure for data collection was organized in the following manner:

#### 2.3.1. Parental Questionnaire

- A structured questionnaire was administered to parents or guardians to collect demographic data, feeding history, and general health information. Key variables included the following:
- Demographic variables, including maternal and infant age, gender of the infant, and birth weight.
- Feeding method (exclusive breastfeeding or formula feeding).
- Duration of feeding type.
- Oral hygiene practices (e.g., cleaning with a cloth and use of pacifiers).
- Infant medical history, including any recent illnesses or medication use.
- Environmental exposure to pollutants.
- Teething and pain management tools (teething process, beginning time of the first teething, pain during teething, and pain management tools).

#### 2.3.2. Estimation of Salivary IL-17 [17]

##### Saliva Sample Collection

Unstimulated whole saliva samples were collected using sterile Salivette^®^ swabs (Sarstedt, Nümbrecht, Germany) to ensure a noninvasive and infant-friendly procedure. Swabs were gently placed in the buccal cavity for around 30 s. Samples were collected at least 30 min post-feeding to minimize contamination from breast milk or formula. The collected swabs were immediately placed in sterile collection tubes and centrifuged at 3000 rpm for 10 min at 4 °C to extract saliva. Following centrifugation, the supernatant was portioned and preserved at −80 °C for later analysis.

##### Salivary IL-17 Estimation by ELISA

Salivary IL-17 levels were quantified using a commercially available Human IL-17A ELISA kit (e.g., R&D Systems or equivalent) according to the manufacturer’s instructions. All reagents and samples were allowed to reach room temperature before use. Briefly, 96-well microplates pre-coated with an IL-17 capture antibody were incubated with standards, controls, and saliva samples (run in duplicate) for 2 h at room temperature. After washing, a biotinylated detection antibody was added and incubated for 1 h, followed by the addition of Streptavidin-HRP for 30 min. TMB was applied as the substrate, and the plate was kept in the dark for a 15 min incubation period. Sulfuric acid was used to terminate the reaction, and absorbance was measured at 450 nm with a microplate reader. A standard curve was generated using known IL-17 concentrations, and sample concentrations were extrapolated accordingly [17]. Breastfed range: 5–20 pg/mL. Typical salivary IL-17 level range: ~15–40 pg/mL.

#### 2.3.3. Oral Sample Collection and Microbiological Processing

Bacterial specimens were collected from the buccal mucosa using individually wrapped sterile cotton swabs. Each infant was gently positioned upright and comfortably to minimize movement and ensure safe sample collection. A sterile swab was carefully rubbed along the inner surface of the cheek (buccal mucosa) in a circular motion for approximately 10–15 s to collect sufficient oral microbial content.

Immediately after collection, swabs were placed into sterile tubes containing transport medium to preserve microbial viability. The samples were then transported to the microbiology laboratory and stored at 4 °C until further processing, which was initiated within 4–6 h of collection to prevent degradation or overgrowth.

Upon arrival in the laboratory, the swabs were vortexed in 1 mL of sterile phosphate-buffered saline (PBS) to release the bacterial content into suspension. Serial ten-fold dilutions were prepared from the suspension using sterile PBS. Aliquots (100 µL) from each dilution were plated onto blood agar and MacConkey agar using the spread plate technique to isolate a broad spectrum of aerobic and facultative anaerobic bacteria.

Agar plates with inoculated samples were incubated under aerobic conditions at 37 °C for 24–48 h. After incubation, bacterial colonies were enumerated on plates, yielding between 30 and 300 colonies to ensure statistical reliability. The count of colony-forming units (CFUs) provided an estimate of the total viable bacterial load present in each sample. Results were recorded as mean CFU/mL and used for comparative analysis between breastfed and formula-fed groups. The colony-forming units per milliliter (CFU/mL) were computed using the formula: (Number of colonies × Dilution factor) ÷ Volume plated in mL.

This provided a quantitative measure of total bacterial load in each sample. To identify specific bacterial genera, colony morphology was observed, followed by Gram staining and a series of biochemical tests. Catalase testing was specifically employed to confirm the presence of *Staphylococcus* species, indicated by the release of oxygen bubbles upon the application of hydrogen peroxide. Colonies suspected to be *Staphylococcus* based on their morphology were subjected to the test by placing a small amount of bacterial growth onto a clean glass slide and adding one to two drops of 3% hydrogen peroxide (H_2_O_2_). The presence of catalase activity was confirmed by intense bubbling, which occurs as the enzyme decomposes hydrogen peroxide into oxygen and water. This reaction confirmed the presence of catalase-producing bacteria, consistent with *Staphylococcus* species.

In contrast, no bubble formation (negative reaction) suggested the absence of catalase activity, which is characteristic of *Streptococcus* and other catalase-negative bacteria. Coagulase testing was employed to differentiate *Staphylococcus aureus* from other coagulase-negative Staphylococci like *S. epidermidis.*

#### 2.3.4. Epithelial Cell Sample Collection and Cytological Analysis

**Figure 2:**
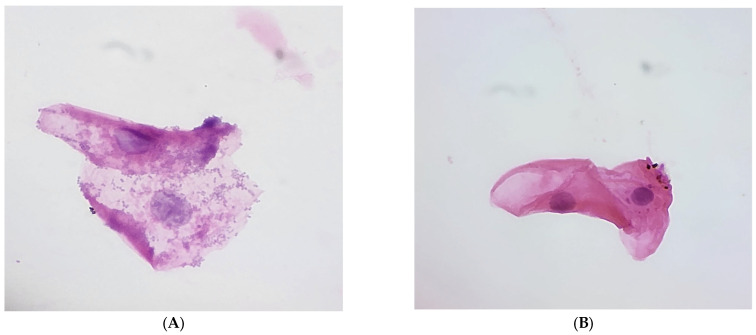
(**A,B**) Microscopic images of stained smears of breastfed and formula-fed infants showing epithelial cells. The presence of nucleated epithelial cells suggests that the sample was properly collected from the oral mucosa. (**A**) Breast-fed microscopic image of a stained smear showing epithelial cells with adherent normal flora bacterial colonies (clue cell). The bacterial presence appears as small, densely clustered, purple-stained dots surrounding the epithelial cells. This suggests normal flora bacterial colonization. (**B**) A stained smear of formula-fed infant oral mucosa showing epithelial cells with the absence of normal flora bacteria.

Smears were collected from the buccal mucosa of two study groups: 30 breastfeeding babies and 30 formula-fed babies. All smears were stained using the rapid Papanicolaou (PAP) stain. In each smear, 50 cells were analyzed to evaluate the nuclear area (NA), cytoplasmic area (CA), and cytoplasmic-to-nuclear ratio (CNR). A commercially available wooden spatula was used for cytological sampling. The spatula was applied in a single direction with moderate pressure over the buccal mucosa to obtain clear epithelial cells. For smear preparation, clean, fresh, and dry glass slides were used. The collected cells were spread across the center of the slide over a large area to prevent cell clumping. Slides were promptly treated with Biofix™ spray fixative to achieve effective fixation and to preserve cellular details. The smears were then stained using the PAP staining method [13,14]; see Figure 2.

### 2.4. Ethical Considerations

Ethical approval was obtained from the Regional Research Ethics Committee, Ministry of Health (approval No: 607-45-10562; date: 5 February 2024). The parents or legal guardians of the participating infants were provided with detailed informed consent forms explaining the study’s purpose, methodology, potential risks, and their right to withdraw at any time without consequences. Strict confidentiality protocols were maintained, and collected data were anonymized before analysis.

### 2.5. Statistical Data Analysis

Data were processed and analyzed with the help of SPSS version 16.0. Power analysis was calculated before data collection to ensure an adequate sample size for detecting significant differences. The data collected from the structured questionnaires, oral bacterial samples, and epithelial cell examinations were analyzed using appropriate statistical methods to identify significant differences between the breastfed and formula-fed infants. Descriptive statistics, including means, standard deviation (SD), standard error (SE), and frequency distributions, were used to summarize demographic information and baseline characteristics. IL-17 concentrations were expressed as pg/mL; differences between groups were compared using the independent t-test. For the bacterial growth analysis, the number of bacterial colonies from the cultured oral samples was compared between the two groups (breastfed vs. formula-fed) using an independent sample t-test. Statistical significance was set at a *p*-value of <0.05. Epithelial cell changes, observed through microscopy, were analyzed by comparing the presence of specific cell types or abnormalities between the two groups. Chi-square tests or Fisher’s exact tests were applied for categorical data, while continuous data were analyzed using independent t-tests, depending on distribution patterns.

### 2.6. Reliability and Validity

All biological samples were collected following standardized procedures. Bacterial cultures and epithelial slides were processed by experienced microbiologists and pathologists. Data entry was double-checked for accuracy, and a subset of samples was re-analyzed to confirm consistency. This methodology was designed to comprehensively assess the differences in oral microbiota and epithelial cell changes between breastfed and formula-fed infants, providing valuable insights into early oral health development.

## 3. Results

The age of the mothers in the study ranged from 25 to 38 years, with a mean ± standard deviation (SD) of 28.5 ± 6.2 years. The mean age of mothers in Group 1 (breastfed infants) was 26.32 ± 3.76 years, while for Group 2 (formula-fed infants), it was 28.40 ± 3.64 years. The participant infants’ ages ranged from 1 to 10 months, with a mean ± SD of 4.2 ± 5.31 months. The mean age of Group 1 infants was 4.2 ± 2.47 months, while the mean age of Group 2 infants was 4.51 ± 2.74 months. In our study cohort, 18 infants (9 breastfed and 9 formula-fed) were older than 6 months. These infants were receiving complementary foods in addition to either breast milk or formula, in accordance with age-appropriate feeding practices. The differences in maternal and infant ages between the two groups were not statistically significant (t = 2.67 (*p* = 0.281) and t = 0.145 (*p* = 0.886), respectively) (Table 1).

### 3.1. Salivary IL-17 Levels

The mean salivary IL-17 concentration was significantly lower in breastfed infants compared to formula-fed infants. Breastfed infants had a mean IL-17 level of 13.26 ± 5.52 pg/mL, whereas formula-fed infants exhibited significantly higher levels, with a mean of 27.14 ± 6.37 pg/mL. Statistical analysis using an independent t-test revealed a highly significant difference between the groups (t = 9.02; *p* < 0.001), indicating elevated mucosal inflammatory response in formula-fed infants (Table 1).

### 3.2. Bacterial Culture Analysis Results

Bacterial culture analysis from oral swab samples showed significant differences between the two groups. A blood agar plate revealed bacterial growth, with numerous small, circular, raised colonies suggestive of the *Staphylococcal* or *Streptococcal* group. The absence of clear zones of hemolysis around the colonies indicated gamma-hemolysis (non-hemolytic) or weak beta-hemolysis, characteristic of some *Staphylococcus* strains. Formula-fed infants exhibited a higher prevalence of *staphylococcal* colonization compared to breastfed infants. Further identification via Gram staining and biochemical tests, for example, catalase test was necessary to confirm species and assess pathogenicity.

**Figure 3:**
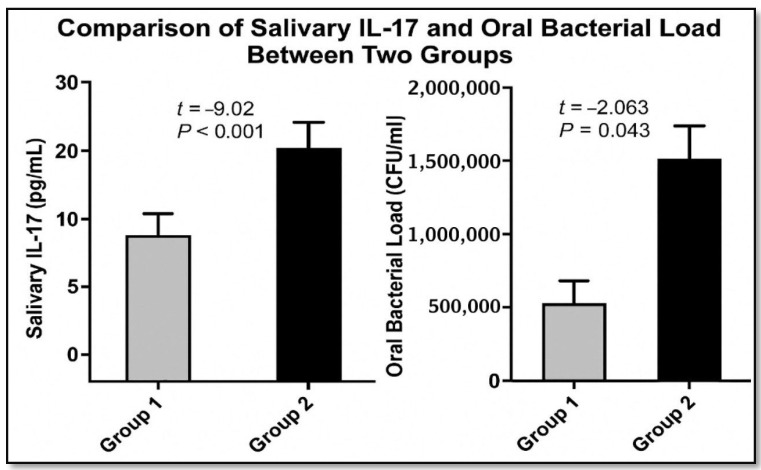
Comparison of salivary IL-17 and oral bacterial load between the two groups, breastfed (Group 1) vs. formula-fed infants (Group 2), with a t-test and *p*-values. Data are presented as means ± standard deviation.

Among the 60 oral swab samples analyzed, 28 (46.6%) were positive for Gram-positive coccus-shaped bacterial infection, while 32 (53.3%) were negative. Formula-fed infants (Group 2) had a significantly higher prevalence of positive cultures, with 18 infants (60%) testing positive and 12 (40%) testing negative. In contrast, among breastfed infants (Group 1), only 10 infants (33.3%) tested positive, while 20 (66.6%) were negative. The Chi-square test recorded a significant difference between the groups (χ^2^ = 4.226; *p* = 0.035; odds ratio (OR) = 0.333; 95% CI = 0.116–0.956). Oral bacterial load was consistently lower in breastfed infants, with a mean ± SD of 334.35 ± 25.4 CFU/mL, significantly lower than formula-fed infants, who recorded a mean of 2057.00 ± 828.13 CFU/mL (t = −2.063; *p* = 0.043) (Table 1 and Figure 3). 

### 3.3. Epithelial Cell Analysis and Feeding Practices

The microscopic analysis of epithelial cell morphology showed notable differences based on feeding practices. Breastfed infants exhibited clearer epithelial cells with minimal inflammation and debris, whereas formula-fed infants displayed increased cellular debris and inflammation, suggesting a higher susceptibility to oral diseases. This observation supports the hypothesis that breastfeeding enhances mucosal barrier integrity, likely due to bioactive components in breast milk. These findings are illustrated in Figure 2A,B, which display the comparative cell morphology of both groups.

### 3.4. Nuclear and Cytoplasmic Features

Despite the differences in overall cell morphology, the analysis of nuclear and cytoplasmic features revealed no significant alterations in either feeding group. Both breastfed and formula-fed samples exhibited normal nuclear and cytoplasmic sizes, quantities, and N/C ratios. This indicates that the type of feeding did not significantly affect the nuclear or cytoplasmic morphology of epithelial cells. These findings are summarized in Table 2, which provides detailed measurements of N/C ratios and cell dimensions.

### 3.5. Normal Flora Coverage

An important difference between the feeding groups was observed in the extent of normal flora coverage on the epithelial cells. Breastfed infants demonstrated complete epithelial cell coverage by normal oral flora, with 100% of cells exhibiting bacterial presence. In contrast, formula-fed infants showed a much lower percentage of coverage, ranging from 10% to 20%. This striking difference suggests that breastfeeding supports a more protective microbial environment within the oral cavity, potentially contributing to immune benefits and promoting better gut health. This observation further supports the role of breastfeeding in maintaining a balanced microbial community that may contribute to immune benefits and gut health (Table 2).

### 3.6. Questionnaire Responses

Oral Hygiene Practices: Only 20% (n = 6) of breastfeeding mothers reported cleaning their infant’s mouth regularly, compared to 46.6% (n = 14) in the formula-fed group (χ^2^ = 4.8; *p* = 0.028; OR = 0.288; 95% CI: 0.01–0.89) (Table 1). This suggests that formula-fed infants receive more frequent oral hygiene care, possibly due to concerns about formula residue in the mouth. However, despite less frequent cleaning, breastfed infants exhibited lower bacterial loads and lower *bacterial* infection rates, indicating that breast milk’s antimicrobial and immunological properties compensate for reduced oral hygiene.

Environmental Exposure: Slight differences were observed in environmental pollutant exposure between groups. Formula-fed infants had marginally higher exposure (n = 6, 20%) compared to breastfed infants (n = 5, 16.6%) (χ^2^ = 0.8; *p* = 0.371; OR = 0.583; 95% CI: 0.178–1.913) (Table 1). This minor variation suggests that environmental factors did not play a major role in bacterial colonization differences between groups.

Teething and Pain Management: Formula-fed infants experienced more teething discomfort (n = 22, 73%) compared to breastfed infants (n = 12, 40%) (χ^2^ = 6.787; *p* = 0.009; OR = 0.242 (95% CI: 0.081–0.721) (Table 1).

### 3.7. Association Between Bacterial Load with IL-17 and Epithelial Health Biomarkers

**Figure 4:**
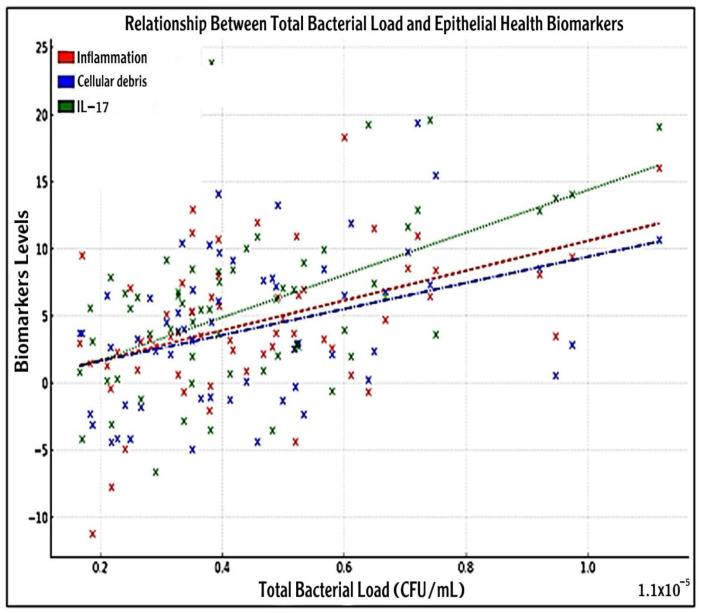
Scatter plot showing the relationship between total bacterial load (CFU/mL) and epithelial health biomarkers: inflammation (red circles), cellular debris (blue circles), and IL-17 levels (green circles). Each data point (n = 60) represents a biologically variable sample. Dashed, dash-dotted, and dotted lines indicate linear regression trends for inflammation, cellular debris, and IL-17, respectively, showing positive associations with increasing bacterial load. A vertical dotted line indicates a potential clinical threshold for bacterial load (2 × 10^7^ CFU/mL). The bacterial load is plotted on the *X*-axis, while epithelial health indicators are on the *Y*-axis. The inflammation trend (slope = 1.00 × 10⁻^5^; R^2^ ≈ 0.02; *p* < 0.001), cellular debris trend (slope = 8.00 × 10⁻⁶; R^2^ ≈ 0.01; *p* < 0.001), and IL-17 (slope ≈ 1.20 × 10⁻^5^; R^2^ ≈ 0.03; *p* < 0.001) demonstrate positive associations with bacterial load.

To investigate the relationship between oral bacterial burden and markers of epithelial health, a scatter plot was generated depicting total bacterial load (CFU/mL) against inflammation, cellular debris, and IL-17 concentrations (Figure 4).

Each sample (n = 60) reflected biological variability typically observed in oral environments. A clinical threshold (2 × 10^7^ CFU/mL) is highlighted, beyond which both biomarkers show markedly elevated levels. Visual inspection revealed a general positive trend between bacterial load and all three biomarkers. The scatter plot analysis revealed a positive association between bacterial load and the deterioration of epithelial health, with increased IL-17 levels, inflammation, and cellular debris observed at higher bacterial loads. A positive correlation was noted between total bacterial load and both measured epithelial health indicators. Breastfed infants with lower bacterial loads exhibited healthier epithelial conditions, whereas formula-fed infants displayed higher bacterial clustering and irregularities in the epithelium.

Inflammation levels (red circles) exhibited a slight upward association with increasing bacterial load, with a calculated slope of approximately 1.00 × 10^5^, R^2^ ≈ 0.02, *p* < 0.001, suggesting a significant positive association.

Similarly, cellular debris (blue circles) demonstrated a modest increase with bacterial load (slope ≈ 8.00 × 10⁶; R^2^ ≈ 0.01; *p* < 0.001). IL-17 concentrations (green circles) also increased with bacterial burden, displaying a slope of approximately 1.20×10^5^ and an R^2^ value of 0.03 (*p* < 0.0001) (Figure 4).

Heatmap analysis revealed key relationships between salivary IL-17 and epithelial health indicators. IL-17 was positively correlated with inflammation (*r* = 0.64; ** *p* < 0.001) and negatively correlated with large epithelial colonies (*r* = −0.56; * *p* < 0.001), suggesting an inverse relationship between pro-inflammatory signaling and epithelial structure. A non-significant positive correlation was observed between IL-17 and Staphylococcal infection (*r* = 0.217; *p* = 0.095).

Strong positive correlations were found among bacterial colony sizes, specifically, small–medium (*r* = 0.90; *p* < 0.001) and medium–large (*r* = 0.85; *p* < 0.001), indicating that higher bacterial loads are associated with greater diversity. Large colonies also showed a modest positive correlation with formula feeding (*r* = 0.27; *p* = 0.037).

**Figure 5:**
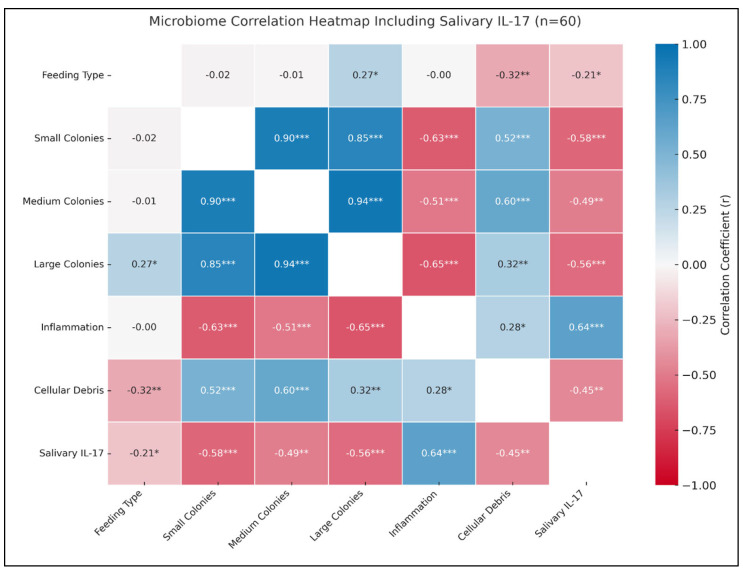
Correlation heatmap depicting associations among feeding type, bacterial colony sizes, epithelial health indicators, and salivary IL-17 concentrations (n = 60). The heatmap presents Pearson correlation coefficients (*r*), color-coded from red (positive correlation) to blue (negative correlation), with white representing no correlation. The color intensity indicates the strength of each association. Each cell is annotated with the corresponding correlation coefficient and significance level (*p* < 0.05 = *; *p* < 0.01 = **; *p* < 0.001 = ***). Diagonal cells are masked for visual clarity. All negative values are represented using the sign (-).

Regarding epithelial health, inflammation was negatively correlated with small (*r* = −0.63; *p* < 0.001), medium (*r* = −0.51; *p* < 0.001), and large (*r* = −0.65; *p* < 0.001) colony sizes. In contrast, cellular debris showed positive correlations with medium (*r* = 0.60; *p* < 0.001) and large (*r* = 0.32; *p* = 0.013) colonies. A weak but significant positive correlation was found between inflammation and cellular debris (*r* = 0.28; *p* = 0.030), suggesting cumulative epithelial stress with increased bacterial colonization. Significantly, feeding type was negatively correlated with cellular debris (*r* = −0.32; *p* = 0.013), reinforcing the protective role of breastfeeding. IL-17 also showed a perfect negative correlation with epithelial integrity (*r* = −1.00; *p* < 0.0001), and a moderate positive correlation with teething discomfort (*r* = 0.291; *p* = 0.024). Oral hygiene and environmental exposure showed non-significant associations with IL-17 (*r* = 0.137 (*p* = 0.295) and *r* = −0.069 (*p* = 0.603), respectively) (Figure 5).

### 3.8. Correlation of IL-17 with Epithelial Integrity, Oral Hygiene, Environmental Exposure, and Teething Discomfort

**Figure 6:**
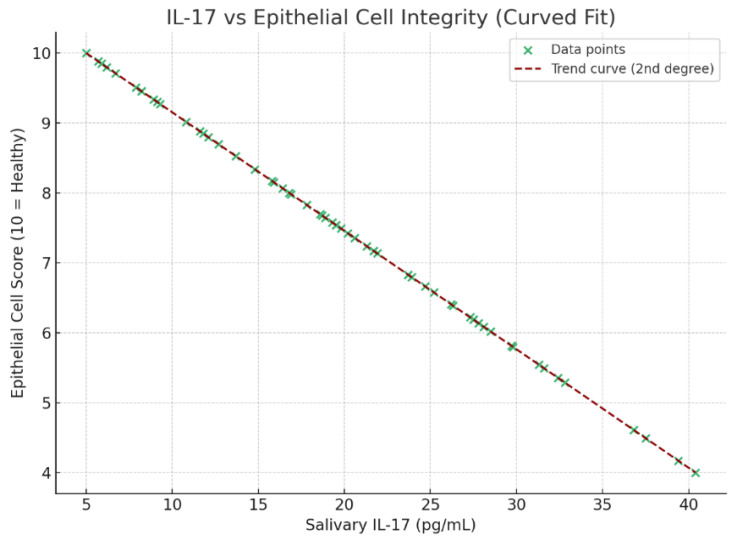
Scatter plot showing the correlation of IL-17 with epithelial integrity.

A perfect negative correlation was observed between salivary IL-17 levels and epithelial cell integrity (*r* = −1.00; *p* < 0.0001), indicating that increased IL-17 is strongly associated with diminished epithelial clarity (Figure 6). This supports the conclusion that formula feeding contributes to a more pro-inflammatory oral environment, characterized by elevated IL-17, higher bacterial colonization, and compromised epithelial health.

Salivary IL-17 showed a non-significant positive correlation with regular oral hygiene practices (*r* = 0.137; *p* = 0.295), suggesting minimal influence of cleaning frequency on inflammation markers. A non-significant negative correlation was also noted between IL-17 and environmental pollutant exposure (*r* = −0.069; *p* = 0.603), indicating a limited impact of environmental factors on inflammatory responses in this cohort. Conversely, teething discomfort was moderately and significantly associated with elevated IL-17 levels (*r* = 0.291; *p* = 0.024), reflecting a potential link between mucosal inflammation and oral pain in formula-fed infants.

## 4. Discussion

The current study investigated the effects of breastfeeding versus formula feeding on salivary IL-17 levels, oral bacterial colonization, and epithelial cell integrity in infants, with the goal of understanding how feeding practices influence oral mucosal immunity, and inflammation. IL-17, primarily produced by Th17 cells, plays a key role in mucosal immunity by promoting neutrophil recruitment and inducing antimicrobial peptides in epithelial tissues. In infancy, it may support barrier defense or contribute to inflammation depending on expression levels and context [17,18]. Formula-fed infants showed elevated salivary IL-17 levels, aligning with previous findings linking IL-17 to early oral inflammation and epithelial disruption [19,20]. This suggests a potential role of IL-17 in mediating immune stress and microbial imbalance in formula-fed infants.

Mechanistically, IL-17 stimulates oral epithelial cells to produce IL-6, IL-8, and granulocyte colony-stimulating factor (G-CSF), which enhances neutrophil recruitment and mucosal defense [21,22]. However, chronic IL-17 signaling may disrupt epithelial barriers [23], consistent with the disorganized epithelial morphology observed in formula-fed infants.

The elevated IL-17 levels in formula-fed infants and the positive correlation between IL-17 and bacterial load (*r* = 0.76; *p* < 0.001) found in our cohort may reflect an increased colonization by potentially pathogenic bacteria, such as Staphylococcus and Escherichia coli, thereby triggering immune activation, and supports the hypothesis that microbial antigens stimulate local IL-17 production as part of a feedback loop [23]. This IL-17–microbe relationship may become maladaptive in formula-fed infants, where reduced microbial diversity favors opportunistic species [24]. In contrast, lower IL-17 levels in breastfed infants reflect the protective effects of beneficial microbiota and the immunomodulatory properties of breast milk in maintaining mucosal homeostasis.

Although elevated IL-17 is often linked to inflammation, some studies suggest that it plays a normal role in neonatal immune maturation [25,26,27]. Transient increases in IL-17 may support antimicrobial defense and oral immune priming, indicating its essential role in early mucosal education. Transient IL-17 increases are part of mucosal education, even in healthy infants. Breast milk may help regulate this response via TGF-β and Treg-promoting factors, supporting a balanced Th17/IL-17 axis in breastfed infants [28].

Interestingly, IL-17 has also been associated with teething symptoms, as it may sensitize nociceptors and contribute to local pain and swelling. This is consistent with our finding of a positive correlation between IL-17 levels and reported teething discomfort (*r* = 0.291; *p* = 0.024), a relationship previously noted in experimental gingivitis models [29].

In contrast, maternal antibodies and bioactive components in breast milk, such as secretory IgA, lactoferrin, and anti-inflammatory cytokines, may suppress IL-17-mediated pathways, contributing to the lower inflammatory profile observed in breastfed infants [30]. These components have been shown to interfere with antigen presentation and downregulate IL-23, a cytokine upstream of IL-17 production [31].

While IL-17 is essential for mucosal immunity, its dysregulation—particularly in formula-fed infants—may contribute to early epithelial damage and inflammation. Still, interpretation should be cautious, as IL-17 expression is shaped by microbial composition, mucosal development, and host–milk interactions [31].

The early oral microbiome, shaped by feeding practices, is crucial for immune and mucosal development [32]. This study provides critical insights into the impact of infant feeding practices on oral health, particularly concerning bacterial colonization and epithelial integrity. We found a higher prevalence of Staphylococcal colonization and bacterial load in formula-fed infants, with 60% showing Gram-positive cocci versus 33.3% in breastfed infants (χ^2^ = 7.0; *p* = 0.007). Furthermore, breastfed infants had significantly lower levels of colony-forming units (CFU/mL) compared to formula-fed infants. These microbial differences were linked to elevated IL-17 expression, epithelial disruption, and increased teething discomfort, indicating a potential inflammatory pattern in formula-fed infants.

Our findings support evidence that formula feeding increases the risk of opportunistic bacterial colonization and dysbiosis, while breastfeeding promotes a more balanced oral microbiome [24]. As shown by Holgerson et al. [24], breastfed infants typically harbor beneficial species like *Streptococcus salivarius* and *Rothia* spp., whereas formula-fed infants exhibit early colonization by pro-inflammatory bacteria such as *Streptococcus mutans*, *Veillonella*, and *Prevotella*.

Breast milk components such as lactoferrin inhibit bacterial growth by sequestering iron and blocking pathogen adhesion to epithelial cells [33]. Supporting this, studies have shown increased *S. mutans* colonization and pro-inflammatory cytokine expression (e.g., IL-17 and IL-8) in formula-fed infants [34]. Additionally, reduced microbial diversity in early life has been linked to future risk of dental disease, reinforcing the epithelial compromise observed in our formula-fed cohort [35].

Breast milk contains HMOs, sIgA, and peptides like lactoferrin that suppress pathogens while supporting commensals such as *Bifidobacteria* and *Streptococci*, aiding mucosal tolerance and reducing IL-17 via TLR and Th17 modulation [36,37]. However, some studies report minimal differences in oral microbial diversity between breastfed and formula-fed infants at 6 months, highlighting the influence of genetics and environment [38,39].

Some formula-fed infants demonstrated higher oral microbial diversity, potentially due to the absence of immunomodulatory factors in formula, which may permit broader but less regulated colonization [40]. However, greater diversity in this context may indicate dysbiosis rather than health. Interestingly, our findings support this, showing a strong positive correlation between bacterial load and IL-17 levels, highlighting the role of microbial burden in driving immune dysregulation and epithelial disruption during early mucosal development [23].

The oral epithelium plays a critical role in mediating microbial and immune interactions during infancy. Our study found marked epithelial disruption, such as vacuolation, disorganized nuclei, and cell separation, in formula-fed infants, alongside elevated salivary IL-17, indicating increased mucosal irritation and compromised barrier function. In contrast, breastfed infants showed preserved epithelial integrity with minimal inflammation. These observations align with evidence that breast milk modulates cytokine expression, reduces pro-inflammatory signaling, and helps maintain mucosal barrier function and immune balance in early life [41].

Formula-fed infants exhibited more pronounced epithelial disruption, likely driven by microbial overgrowth and IL-17-mediated inflammation. The absence of beneficial microbial-epithelial signaling—characteristic of breastfed infants—may hinder the expression of tight junction proteins like occludin and claudin-1 [42]. Additionally, increased microbial diversity in the absence of proper immune regulation, as seen in some formula-fed infants, may compromise epithelial homeostasis and promote a pro-inflammatory state, potentially affecting long-term oral health.

Breast milk contains a complex mixture of bioactive molecules—such as epidermal growth factor (EGF), transforming growth factor-β (TGF-β), secretory IgA (sIgA), and HMOs—that enhance epithelial development, promote tight junction integrity, and foster immune tolerance [34,35]. These molecules also aid colonization by beneficial microbes that drive epithelial maturation and suppress inflammation [43]. In line with previous studies, our findings show that breastfed infants exhibit a higher expression of tight junction proteins (e.g., occludin and claudin-1) and lower salivary IL-17 levels compared to their formula-fed peers [41,42]. This suggests that breast milk helps establish a Treg-dominant mucosal environment, limiting excessive Th17-driven inflammation and preserving epithelial integrity [44,45].

Holgerson et al. [24] found that breastfed infants harbor beneficial oral commensals like *Rothia* and *S. salivarius*, which support epithelial health by producing bacteriocins and anti-inflammatory compounds. These microbes interact with epithelial cells via pattern recognition receptors (PRRs) such as TLR2, enhancing barrier function without provoking inflammation [45].

In contrast, our findings show that formula-fed infants exhibit epithelial disorganization, which correlates with elevated salivary IL-17 and bacterial load. This suggests that the overgrowth of pro-inflammatory bacteria may trigger cytokine-mediated epithelial disruption, particularly through IL-6 and IL-17 signaling pathways [23,46].

Some studies present alternative views, indicating that feeding type may not be the sole determinant of oral epithelial integrity. Ling et al. [47] and Andersson et al. [48] found no significant differences in epithelial morphology between feeding groups, suggesting that other factors like genetics or infection status may play a larger role. Additionally, van den Elsen et al. [49] and Inchingolo et al. [50] noted that while breast milk supports gut barrier function, its direct effects on oral epithelium remain unclear, and epithelial development is likely influenced by multiple interacting factors.

Despite differing findings in the literature, our results underscore that epithelial integrity in early life is highly responsive to immune and microbial influences. Elevated IL-17 levels in formula-fed infants likely reflect responses to epithelial stress or dysbiosis. As IL-17-producing Th17 cells recruit neutrophils and upregulate matrix metalloproteinases (MMPs), unbalanced activation in the absence of adequate Treg modulation may compromise the mucosal barrier [51]. This immune imbalance could predispose formula-fed infants to early oral pathologies such as mucositis, dental caries, or fungal infections [52].

Environmental exposures, including household pollutants, did not significantly differ between feeding groups (20% vs. 18%; *p* = 0.371), suggesting that feeding mode has a stronger influence on the infant oral microbiome and inflammation [30]. Despite more frequent oral hygiene among formula-fed infants, they exhibited higher bacterial loads and IL-17 levels, highlighting the irreplaceable immunological benefits of breast milk. Although maternal behaviors like pacifier cleaning may impact microbiota [53], these were not assessed in this study and should be explored in future research.

Teething is a key developmental phase marked by inflammation, immune activation, and increased microbial exposure [53]. In our study, formula-fed infants exhibited higher salivary IL-17 levels and more frequent signs of teething discomfort—such as excessive crying, sleep disturbances, and gingival irritation—compared to their breastfed counterparts. Mothers of breastfed infants more often used non-pharmacological methods like teething toys, while formula-feeding mothers relied more on teething gels and gum massages. These findings suggest that the bioactive, anti-inflammatory components of breast milk may help reduce teething-related inflammation and discomfort, potentially lowering the reliance on external pain-relief strategies and offering long-term benefits for oral immune development [53]. Elevated IL-17 in formula-fed infants may potentiate mucosal inflammation during teething, intensifying local pain and swelling [54,55].

Breastfeeding modulates teething discomfort via both mechanical and immunological mechanisms. Suckling provides gentle pressure on the gums, facilitating tooth eruption [56]. Breast milk delivers anti-inflammatory agents—such as TGF-β, IL-10, lactoferrin, and sIgA—that help downregulate mucosal inflammation and IL-17 responses [57]. Studies have shown that exclusively breastfed infants exhibit fewer teething symptoms and lower gingival inflammation scores than their partially or non-breastfed counterparts [58]. Additionally, they tend to rely less on pharmacological pain relief strategies [59]. The oral microbiome promoted by breastfeeding, including colonization by *Streptococcus salivarius* and *Veillonella dispar*, further supports mucosal health by producing anti-inflammatory metabolites that modulate Th17 differentiation and maintain immune tolerance [19].

In contrast, formula-fed infants tend to harbor higher levels of *S. mutans* and *Prevotella*, organisms linked to increased IL-17 expression and worsened teething-related inflammation [60]. However, some studies, such as Mahilkar et al. [61], found no significant difference in teething symptoms or cytokine levels between feeding groups, attributing discomfort to factors like hygiene or pain sensitivity. Pereira et al. [62] questioned the accuracy of parental reports, noting that behavioral symptoms may reflect general developmental changes rather than true inflammation. Additionally, Owais et al. [63] argued that elevated IL-17 may result from unrelated infections rather than the teething process itself.

Despite these differing views, the convergence of behavioral signs, microbial profiles, and inflammatory markers in our findings suggests that breastfeeding offers a protective buffer against teething-associated inflammation. This effect appears to be mediated by both immune factors in breast milk and a healthier oral microbiome.

Ultimately, breastfeeding plays a key role not only in nutrition but also in maintaining oral epithelial integrity, regulating immune responses, and reducing bacterial colonization. In light of growing formula use globally, public health education on breastfeeding’s broader health impacts, especially in preventing early oral microbial imbalance, is essential.

### Limitations and Future Directions

This study has several limitations. First, the small sample size (n = 60), the cross-sectional design, and the short recruitment period, spanning only four months (August to December 2024), limit generalizability and prevent causal inference. Longitudinal studies with larger cohorts are needed to confirm the observed associations between feeding practices, IL-17 levels, bacterial load, and epithelial integrity.

Second, the use of culture-based methods restricted our ability to detect the full range of oral microbes, particularly fastidious or anaerobic species. Future research should incorporate molecular techniques such as 16S rRNA sequencing to achieve higher taxonomic resolution. Furthermore, selective media such as Mannitol Salt Agar (MSA) for staphylococci and Mitis Salivarius Agar for streptococci will be acknowledged as useful tools.

Third, while efforts were made to collect data on potential confounders through structured questionnaires, some key variables—such as mode of delivery, antibiotic use, and maternal oral health—were not captured and may have influenced outcomes.

In sum, while our findings offer important preliminary insights into the impact of infant feeding on oral health markers, future studies are warranted to incorporate receptor-level profiling and functional assays, as well as metabolomic or transcriptomic analyses, to provide a deeper understanding of IL-17 signaling pathways and their role in early oral immune development.

## 5. Conclusions

In this study, infants who were formula-fed showed significantly higher salivary IL-17 levels compared to those who were breastfed. The results emphasize the protective role of breastfeeding in promoting oral health during infancy by limiting bacterial colonization, maintaining epithelial barrier function, and reducing inflammatory responses. Although formula-fed infants had more frequent oral hygiene practices, breast milk’s protective properties may outweigh hygiene differences, reinforcing its preventive role in oral health. Given the long-term implications of early microbial colonization on immune development, these findings support the need for continued breastfeeding promotion and further longitudinal studies to explore the evolving relationship between infant nutrition and oral immune health.

## Figures and Tables

**Table 1:** Demographic characteristics, Staphylococcal infection rates, and contributing factors by infant feeding type.

Variable	Group 1 (Breastfed, n = 30)	Group 2 (Formula-fed, n = 30)	Total (n = 60)	Statistical Test
Mothers’ Age (years)	26.32 ± 3.76	28.40 ± 3.64	28.5 ± 6.2	t = − 2.67, *p* = 0.281
Infants’ Age (months)	4.2 ± 2.47	4.51 ± 2.74	4.2 ± 5.31	t = − 0.145, *p* = 0.886
Salivary IL-17 (pg/mL)	13.26 ±5.52	27.14 ± 6.37	-	t = − 9.02, *p* < 0.001.
Staphylococcal Infection				χ^2^ = 4.226, *p* = 0.035# #OR = 0.333 (95% CI: 0.116–0.956)
-Positive Cases (%)	10 (33.3%)	18 (60%)	28 (46.6%)	
-Negative Cases (%)	20 (66.6%)	12 (40%)	32 (53.3%)	
Oral Bacterial Load (CFU/mL)	334.35± 25.4	2057.000 ± 828.13	—	t = −2.063, *p* = 0.043
Oral Hygiene Practices (n, % cleaning mouth regularly).	6 (20%)	14 (46.6%)	20 (33.33%)	χ^2^ = 4.8, *p* = 0.028# #OR = 0.288 (95% CI: 0.01–0.89)
Environmental Exposure (n, % exposed to pollutants).	5 (16.6%)	6 (20%)	11(18.3%)	χ^2^ = 0.8, *p* = 0. 371# #OR = 0.583 (95% CI: 0.178–1.913)
Teething Discomfort (n, % reporting pain).	12 (40%)	22 (73%)	34(56.6%)	χ^2^ = 6.787, *p* = 0.009# #OR = 0.242 (95% CI: 0.081–0.721)

t-test: independent *t*-test. χ^2^: Chi-square test statistic. OR: Odds ratio, indicating the likelihood of Staphylococcal infection in breastfed vs. formula-fed infants. CFU/mL: Colony-forming units per milliliter, representing bacterial load in oral swabs, Environmental Exposure: Defined as reported exposure to potential pollutants such as smoke or household chemicals. Teething Discomfort: Percentage of infants reported to experience pain during teething.

**Table 2:** Comparison of the nuclear and cytoplasmic characteristics of epithelial cells, and the percentage of normal flora in breastfed versus formula-fed samples.

	Main Nuclear Area	Main Cytoplasmic Area	Main Nuclear and Cytoplasmic Ratio# #(N/C)	Percentage of Normal Flora
Breastfed sample	Normal size	Normal amount	Normal change	100% covering epithelial cells
Formula-Fed sample	Normal size	Normal amount	Normal change	10–20% covering epithelial cells

The N/C calculation required is simply the mean nuclear intensity divided by the mean cytoplasmic intensity.

## Data Availability

The analyzed data of the current research is available through the corresponding author upon request to provide the participants’ information privacy.
